# A Dual Coordinate System Vertebra Landmark Detection Network with Sparse-to-Dense Vertebral Line Interpolation

**DOI:** 10.3390/bioengineering11010101

**Published:** 2024-01-22

**Authors:** Han Zhang, Albert C. S. Chung

**Affiliations:** Department of Computer Science and Engineering, The Hong Kong University of Science and Technology, Clear Water Bay, Hong Kong

**Keywords:** computer-aided diagnosis, convolutional neural network, vertebra landmark detection, scoliosis assessment

## Abstract

Precise surveillance and assessment of spinal disorders are important for improving health care and patient survival rates. The assessment of spinal disorders, such as scoliosis assessment, depends heavily on precise vertebra landmark localization. However, existing methods usually search for only a handful of keypoints in a high-resolution image. In this paper, we propose the S2D-VLI VLDet network, a unified end-to-end vertebra landmark detection network for the assessment of scoliosis. The proposed network considers the spatially relevant information both from inside and between vertebrae. The new vertebral line interpolation method converts the training labels from sparse to dense, which can improve the network learning process and method performance. In addition, through the combined use of the Cartesian and polar coordinate systems in our method, the symmetric mean absolute percentage error (SMAPE) in scoliosis assessment can be reduced substantially. Specifically, as shown in the experiments, the SMAPE value decreases from 9.82 to 8.28. The experimental results indicate that our proposed approach is beneficial for estimating the Cobb angle and identifying landmarks in X-ray scans with low contrast.

## 1. Introduction

Spinal deformation [1] is a severe orthopedic symptom that can significantly affect the functioning of the heart and lungs. Among various spinal deformities, scoliosis is particularly prevalent and can have long-term detrimental effects on health if left untreated. Therefore, the evaluation of idiopathic scoliosis is crucial for accurate diagnosis. The Cobb angle [2], which measures the spine’s bending angle on X-ray views, is the gold standard for assessing idiopathic scoliosis in clinical practice. Clinicians typically measure the Cobb angle manually by identifying landmarks and using straight lines to determine the angle.

However, this manual technique has drawbacks, such as imprecise landmark locations and the time-consuming annotation of X-ray images. Four landmarks on each vertebra are first detected, and then a quadrilateral bounding box is used to connect them. Subsequently, a straight line extends along each vertebra’s lower or upper edge of the border. One angle can be generated for each pair of straight lines with different quadrilaterals. When these two lines are parallel, the Cobb angle is zero. The clinical experts select the maximum of these angles as the final measured Cobb angle. This manual technique of measuring has some drawbacks, however. First, landmark locations can be different and varying since the landmarks are selected based on clinicians’ subjective judgment, which can also be affected by the image quality and result in a significant error and uncertainty [3]. In addition, annotating the X-ray images is time-consuming since it comprises multiple vertebrae (at least five lumbar and twelve thoracics). The clinicians may have to perform multiple measurements to determine the optimal Cobb angle. To get more precise measurements, clinicians often choose the most suitable landmarks based on their knowledge and experiences, which can vary among different clinicians. For these reasons, Cobb angle computations need to be automated.

Some recent research works have demonstrated reliable performances for the automated Cobb angle measurement task, as will be discussed in Section 2. However, researchers have not thoroughly investigated the inter-relationships and characteristics between vertebrae, nor have they integrated domain knowledge into the network architecture construction. The anterior–posterior spinal X-ray scans exhibit a disparity in background interference between the upper and lower regions. The upper area demonstrates less presence of additional components, and the lower area may be obscured by surrounding tissues. Hence, it is challenging to localize accurately. In this situation, relying exclusively on regional information is inadequate for accurately localizing vertebral landmarks, especially in the lower area. Nevertheless, none of the aforementioned methods take into consideration the positional relationship between vertebrae that can be derived from current annotations without the requirement for additional annotations.

In this paper, we introduce the sparse-to-dense vertebral line interpolation vertebra landmark detector (S2D-VLI VLDet) network. This end-to-end network has the capability of considering the correlation between vertebral structures and landmarks and effectively integrates dual coordinate systems into a unified framework. An S2D-VLI VLDet network can alleviate these abovementioned issues by using adjacent center point interval offsets and adjacent vertebra interval offsets as supplementary supervision information. The effectiveness of the ground truth is enhanced through the integration of multiple coordinate systems that are tailored to the specific sub-tasks. Finally, by introducing the vertebral line interpolation scheme, the design of the ground truth can be more reasonable and practical by improving the network training process.

Overall, our proposed method has the following contributions:

- We introduce an effective scheme, namely the center point interval estimator (CPIE), as an approach to obtain inter-vertebral supervision information to estimate the center points of vertebrae. The proposed scheme enhances the precision of center point localization, particularly in cases where vertebrae are subject to significant background interference. This improvement effectively reduces the accumulation of errors caused by inaccurately identified center points.
- We introduce the implementation of an auxiliary task, namely the adjacent vertebra interval estimator (AVIE). This task aims to effectively utilize the implicit knowledge the existing annotations provide.
- We introduce a novel approach using a dual coordinate system during the learning process. Our strategy involves the utilization of both Cartesian and polar coordinate systems for presenting the ground truth of center points and corner offsets, respectively. The sub-tasks can effectively preserve the advantages to a greater extent by using multiple coordinate systems.
- We introduce a vertebral line interpolation scheme to alleviate the drawbacks of the ground truth design during the network training process by converting the ground truth from sparse to dense.
- We propose a novel evaluation metric named self-adaptive MDE to analyze the sources of errors better. These include the misordering of vertebral pairs and inaccurate localization of the landmarks under correct ordering.

This research work is an extension of our previous work presented in [4]. The main differences between the current manuscript and our previously published work are as follows. First, this manuscript proposes a new vertebral line interpolation scheme, which can provide more implicit supervisory information. Second, a novel self-adaptive MDE has been proposed to evaluate the vertebra landmark detection results objectively. Third, more extensive experiments have been included to demonstrate the properties of the proposed method.

## 2. Related Work

Landmark detection is a widely studied topic in the computer vision field. Some classic landmark detection frameworks tailored for natural images and videos, such as pose estimation and facial keypoint detection, are also exploited for CADx, such as the vertebra landmark detection task. Deep learning methods have recently been widely utilized for landmark recognition because of their effectiveness and efficiency. This section is arranged as follows. We first present some classic and state-of-the-art landmark detection-related works in Section 2.1. Most of the related work in Section 2.1 has been tailored for natural images and videos, such as pose estimation and facial keypoint detection. In Section 2.2, we introduce some spinal-related landmark detection approaches, which mainly work on medical images for clinical applications.

### 2.1. Landmark Detection

Most of the convolutional neural network (CNN) methods can be broadly divided into two categories: regression and heatmap models.

The abovementioned regression category can be subdivided further into one-stage models that directly conduct the landmark regression task and cascaded regression models [5]. As for the one-stage regression models, using this kind of regression model for the landmark detection task has been widely explored [6,7,8,9,10,11,12,13,14,15]. These one-stage models always extract the landmark coordinates represented by a vector from an input image. The dimension of the vector is twice the number of landmarks since the vector consists of both the x and y coordinates of all landmarks. As for the backbone networks, any network architecture can be used for feature extraction. Unlike the one-stage regression models, which directly identify landmark coordinates, cascaded regression models successively update specified or previously discovered landmarks to detect the landmarks step by step for higher precision [16,17,18,19,20,21,22,23,24]. A sub-network always works to generate a vector for updating the landmark locations step by step. Following the updates, the model produces the final landmark coordinates. Comparing one-stage models to cascade models demonstrates that cascade regression models are often more competitive than the one-stage regression models due to their coarse-to-fine progression [25].

As for the aforementioned heatmap models, by detecting the two-dimensional locations highlighted on the heatmap, the landmarks can be recognized from the heatmap. The design of the heatmap regression model is typically influenced by the fully convolutional network (FCN) proposed by [26]. FCN utilizes a convolutional encoder to extract semantic features from the input image and decodes these features into a heatmap using a deconvolutional decoder. Based on their properties, heat map approaches can be classified into distribution models, heatmap regression models, and pixel classification models [5]. The distribution model describes the positions of landmarks using multivariate distributions. The center of the distribution indicates the coordinates of the landmarks. The landmarks are described using a two-dimensional Gaussian distribution [27,28,29,30]. The numbers of channels of the heatmap and landmarks are the same. The summation of the elements in each heatmap is forced to equal one using a spatial softmax function. In [31], the authors introduced another heatmap regression model for detecting landmarks from heatmaps. While Convolutional Pose Machines (CPMs) are currently utilized for human body landmark recognition, CPMs can be extended to detect landmarks in medical images. The most significant difference between the distribution model and the heatmap regression model is that the heatmap regression model’s output includes a heatmap for the background pixels.

Besides these two kinds of models mentioned above, the authors of Mask R-CNN [32] also studied the detection of landmarks using pixel-wise classification models. Based on this pixel-wise classification model, in [5], the authors proposed a pixel-wise classification model with a discriminator, which can achieve a better performance. Although most of the abovementioned models target the facial landmark detection task, they are easy to exploit in medical images and have heuristic significance for landmark detection in medical images. With the rapid development of transformer-based models in computer vision in recent years, the transformer-based methods also show competitive performance on landmark detection tasks.

In [33], ViTPose, which estimates pose based on the plain vision transformers, exhibits good competence in model structure simplicity, model size scalability, training paradigm flexibility, and model knowledge transferability. It is demonstrated experimentally that the basic ViTPose model is able to outperform its closely related methods in the challenging MS COCO (Microsoft Common Objects in Context) Keypoint Detection benchmark. In addition to a number of single-task models, more powerful models for multiple computer vision tasks are proposed. In [34], it shows that if a shared pixel-to-sequence interface is utilized, a set of different core computer vision tasks can be unified, for example, object detection, instance segmentation, keypoint detection, and image captioning, which have different output types (e.g., bounding boxes or dense masks).

### 2.2. Vertebra Landmark Detection and Automated Cobb Angle Estimation

Much research work has been focusing on the automated calculation of the Cobb angle, which can be broadly classified into two groups: the direct estimation approaches and the indirect estimation approaches. The indirect estimation approaches always achieve landmark detection following two steps. First, the model segments all the vertebrae from the medical scans using the segmentation methods, including some traditional methods or the CNN models [35]. After that, to produce a straight line along the direction of each vertebral edge, some straight line-fitting techniques can be used [36,37]. The Cobb angle is then determined by calculating the largest angle between these pairs of lines. The direct estimation method is identical to the clinical expert’s manual pipeline measurement. The clinicians always select four landmarks on each vertebral body within the medical image. To compute the Cobb angle, the pairs of landmarks are then connected to make a straight line. Therefore, the most important step is to detect all the landmarks accurately.

Both direct and indirect approaches are widely used in current research works for estimating the Cobb angles. The authors in [38] predicted all the landmarks using the proposed structured multi-output regression model. BoostNet, mentioned in [39], creates a CNN model for enhancing the feature from the X-ray images so that landmarks can be localized with greater precision. The authors in [40] considered the knowledge from both AP X-rays and LAT X-rays and consequently designed an MVC-net to simultaneously predict the landmarks and Cobb angle. Wang et al. [41] developed a multi-view extrapolation network for combined learning of landmarks and the Cobb angle and presented an alternate post-processing optimization approach. The authors in [42] took into account the location of vertebral landmarks and developed a two-stage framework to predict the vertebral area and landmarks sequentially. The authors in [43] employed RetinaNet [44] to identify vertebrae, followed by HR-Net [45] to detect four corner points on the vertebra region. Once the landmarks have been discovered, the Cobb angle can be determined directly following the clinical rules. The authors in [46] proposed an MPF-net, combining the vertebra detection branch and the landmark prediction branch to provide the landmark detection task with bounded area information. A landmark detection network on the lumbar X-ray images was proposed by An et al. based on the properties of the X-ray images and shapes of the vertebrae [47]. 304 X-ray images were used for the evaluation of the proposed method. In center detection, the proposed network gave an accuracy of 98.02%, and in corner detection, it gave a relative distance error of 8.34%.

On the other hand, the combined use of landmark detection and vertebra segmentation can indirectly estimate the Cobb angle. But, the detection results can be sensitive to errors in detection and segmentation. In [48], a novel deep-learning architecture, namely the vertebra localization and tilt estimation network (VLTENet), has been proposed. This network employs tilt estimation and vertebra localization as network prediction goals, which improves the accuracy of Cobb angle estimation.

## 3. Method

### 3.1. Vertebra Landmark Detector Framework

**Figure 1:**
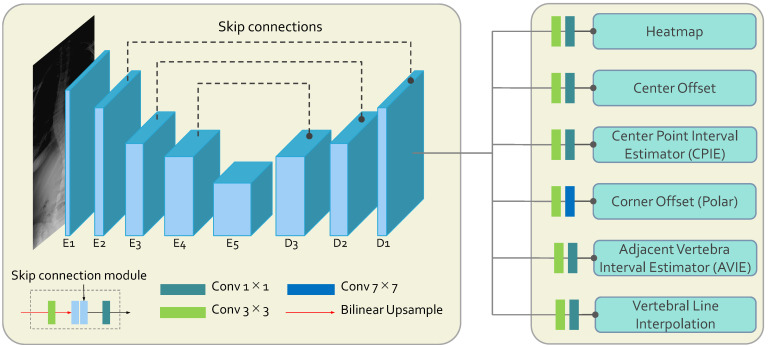
The brief network structure adopted the S2D-VLI VLDet model. The symbols *E* and *D* are used to denote the encoder and decoder, respectively.

The S2D-VLI VLDet network is a novel model utilizing the sparse-to-dense vertebral line interpolation approach and the dual coordinate system. The proposed dual coordinate system modeling replaces the intrinsic coordinate system, the Cartesian coordinate system modeling, which most of the existing vertebra landmark detectors utilize. Overall, as indicated in Figure 1, the whole framework is an encoder–decoder architecture using the entire X-ray images as inputs. The encoder of S2D-VLI VLDet employs a modified pre-trained ResNet34 [49] as its backbone to extract semantic features from X-ray scans. It consists of five convolutional blocks, which are $E_{1}$ to $E_{5}$, as illustrated in Figure 1. The decoder consists of three convolutional blocks ($D_{1}$ to $D_{3}$) and six pathways for decoding. Subsequently, the skip connections incorporate deep and shallow features, denoted using the black-dotted lines between encoder and decoder blocks in Figure 1. As for the decoder of the whole framework, there are six paths for further landmark decoding. The heatmap pathway is employed to estimate the center points of the vertebrae. The center offset and corner offset paths are employed to estimate the Cartesian coordinates of the center points and the polar coordinates of four corner points, respectively. In order to enhance the accuracy of the center point and corner point regression, the S2D-VLI VLDet model has incorporated and implemented two supplementary pathways, namely CPIE and AVIE. As illustrated in Equation (1), there are six loss terms: the heatmap loss ($L_{hm}$), the center offset regression loss ($L_{center}$), the corner offset regression loss ($L_{corner}$), the CPIE loss ($L_{CPIE}$), the AVIE loss ($L_{AVIE}$), and the vertebral line interpolation loss ($L_{VIL}$). Empirically, we ensure all the loss items are similar. Therefore, hyper-parameters $\alpha_{1}$ to $\alpha_{5}$ are all 1, and $\alpha_{6}$ is set to 0.05. They are jointly optimized.

(1) $$\begin{array}{c} L=\alpha_{1}L_{hm}+\alpha_{2}L_{center}+\alpha_{3}L_{corner}\\ +\alpha_{4}L_{CPIE}+\alpha_{5}L_{AVIE}+\alpha_{6}L_{VIL}. \end{array}$$

As stated in [50,51], during the inference phase, an unnormalized two-dimensional Gaussian disc is employed in estimating and recognizing the expected center point based on the highest response observed in the heatmap subsequent to the decoding process. We utilize the focal loss [50] to optimize the model parameters in the center point pathway with identical parameter settings. The main purpose of the center offset pathway is to alleviate the quantization error that arises from downsampling inputs and employing b-spline interpolation. The coordinates of the points in the downsampled feature map are denoted as (⌊h/k⌋, ⌊w/k⌋), where *k* represents the downsampling factor [50] and *h* and *w* denote the height and width of the input images, respectively. The center offset can be mathematically expressed as (h/k − ⌊h/k⌋, w/k − ⌊w/k⌋), and the L1 loss is used to optimize the parameters.

### 3.2. Dual Coordinate System

After extracting the center points using the outputs from the heatmap and center offset pathways, the center points and the corresponding corner offsets determine the four corner points. Each vertebra has four corner offsets, which are vector representations starting from the center and extending towards the four corners. In the polar coordinate system, the polar diameter and the polar angle are essentially the length and angle, representing the point’s orientation involving the distance or angle. Each vertebra has nearly symmetrical characteristics, so the distances between its center and its four corner points are close. As a result, the polar coordinates are utilized to represent the corner points, with the center points serving as the reference origins. This approach allows for a more focused analysis of the angles and distances between the corner and center points. Nevertheless, solely depending on the Cartesian coordinate system would make this information insufficiently explicit for the network to learn. The adoption of the polar coordinate system enhances the network’s ability to differentiate variations in distances and angles, resulting in a more accurate corner point localization procedure. In contrast, the utilization of the Cartesian coordinate system shows superior performance in the center point localization sub-task since it is less reliant on a fixed origin. Therefore, it is more beneficial to employ the Cartesian coordinate system for the purpose of obtaining the precise positions of the center points of the vertebrae. In order to provide enhanced precision in localizing both center points and corner points, different coordinate systems are employed due to the different characteristics between them. The utilization of dual coordinate systems in conjunction can enhance the network’s capacity for learning while preventing the learning performance from worsening due to the irrationality of ground truth coordinates.

**Figure 2:**
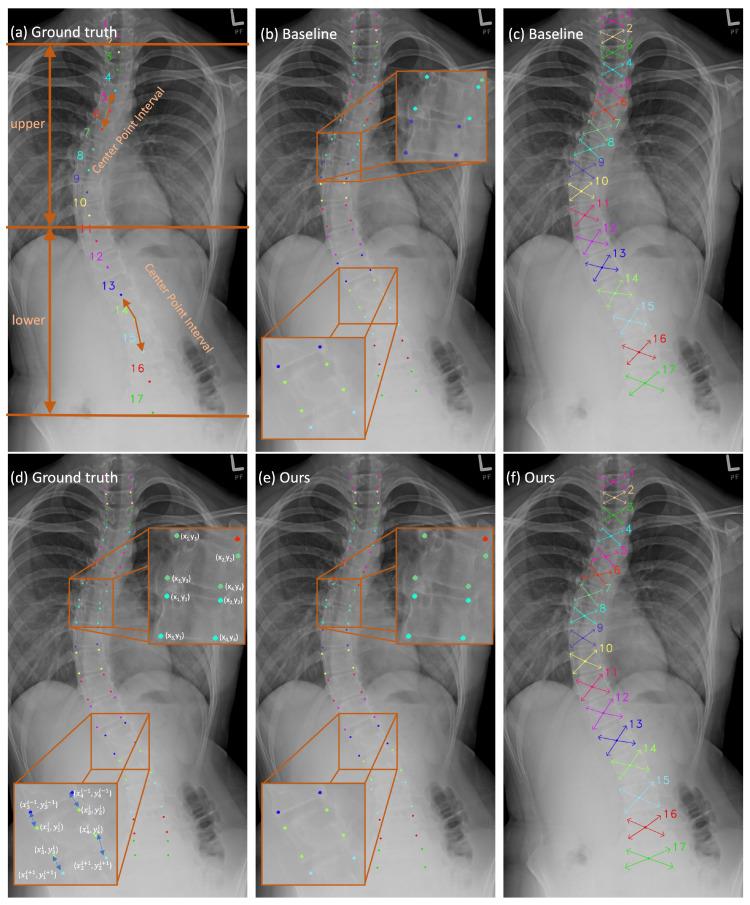
The qualitative results in these figures are generated through the S2D-VLI VLDet model. (**a**,**d**) can be considered ground truths. (**b**,**c**) illustrate the outputs of the baseline approach [50] in terms of vertebra landmark detection. Meanwhile, (**e**,**f**) demonstrate the results of our approach to vertebra landmark detection.

To represent the four corner points of each vertebra in a Cartesian coordinate system, they are usually written as ($x_{1}^{i}$, $y_{1}^{i}$), ($x_{2}^{i}$, $y_{2}^{i}$), ($x_{3}^{i}$, $y_{3}^{i}$), and ($x_{4}^{i}$, $y_{4}^{i}$), as shown in Figure 2d, where *i* denotes the indices of the vertebra, which range from 1 to 17 in the AASCE dataset. To convert them to polar coordinates, we first identify the center of each vertebra as the pole of the polar coordinate system. The orientation of the polar axis in the positive direction is conventionally expressed as the horizontal right direction. Additionally, the polar angle is expressed in radians and is measured anticlockwise. The polar coordinates of the four corners can be denoted as ($r_{1}^{i}$, $\theta_{1}^{i}$), ($r_{2}^{i}$, $\theta_{2}^{i}$), ($r_{3}^{i}$, $\theta_{3}^{i}$), and ($r_{4}^{i}$, $\theta_{4}^{i}$). These coordinates are used as the ground truth for the training process of the corner offset pathway. The optimization of the corner offset pathway can be achieved through the L1 loss. Converting the corner points from the polar coordinates format to Cartesian coordinates is necessary during the inference phase. This is because the performance evaluation of the vertebra landmark localization is more easily conducted under the Cartesian coordinate system. At first, the Cartesian coordinates of the center point ($x_{ct}^{i},y_{ct}^{i}$) are obtained from the outputs derived from the heatmap and the center offset pathway. Then, applying the transformation method, Equation (2) [52], the four corner points in the form [($x_{1}^{i}$, $y_{1}^{i}$), ($x_{2}^{i}$, $y_{2}^{i}$), ($x_{3}^{i}$, $y_{3}^{i}$), and ($x_{4}^{i}$, $y_{4}^{i}$)] can be determined, where *m* represents the indices of each vertebra’s corner points.

(2) $$\begin{array}{c} x_{m}^{i}=x_{ct}^{i}+r_{m}^{i}\cos\left(\theta_{m}^{i}\right),\\ y_{m}^{i}=y_{ct}^{i}+r_{m}^{i}\sin\left(\theta_{m}^{i}\right). \end{array}$$

### 3.3. Center Point Interval Estimator and Adjacent Vertebra Interval Estimator

The spine X-ray scan in Figure 2a reveals a distinct difference in background interference levels between the upper and lower regions of the spine. As seen in Figure 2a, the 17 vertebrae comprising the spine are divided into two separate groups based on the severity of background interference. The first group comprises the top ten vertebrae, while the second comprises the bottom seven vertebrae. This division is defined based on how severe the interference from the background is. The upper region background consists mainly of the lung, whereas the lower half is interfered with by the thoracic and abdominal cavities. Due to the density and morphology of the tissue and organ, the background interference is more severe in the lower half. As a result, it is more difficult to find the vertebrae in the lower part. The mean detection error (MDE), which is computed in pixels, can yield more precise quantitative findings for locating 17 center points and 68 landmarks. The results can be found in Table 1. The baseline approach yields a significantly higher MDE (approximately 50%) for center points in the lower region compared to the upper region, resulting in an overall MDE increase of approximately ten pixels for the entire 17-vertebrae spine. Meanwhile, the accumulation of errors in the localization of the center points will result in inaccuracies in the localization of the corner points. Consequently, this will lead to errors in the calculation of the Cobb angle, which is used for the assessment of scoliosis. As a result, to improve the precision of center point localization, we propose the CPIE approach, which incorporates adjacent center point intervals, which are indicated by double-sided arrows in Figure 2a. Once the additional pathway (CPIE pathway) is incorporated as a supplementary pathway following the decoder, it enables a comprehensive analysis of the correlation between the center points in neighboring vertebrae. This correlation actively contributes to the training process through the back-propagation process. Furthermore, by employing a multi-task learning scheme as a regularization strategy that incorporates an inductive bias [53], potential bias towards specific tasks during training is mitigated, thereby reducing the risk of overfitting [53].

The primary reason for the overall error results from the inconsistency between the predicted corner points and their practical location. Instead of being situated at the border corners of the vertebrae, the predicted corner points are found within the interior of the vertebrae, as shown in Figure 2b. It demonstrates that the estimated corner points obtained using the baseline method lack sufficient precision. The fact that the border between two neighboring vertebrae is not properly differentiated is a possible explanation for this finding that makes intuitive sense. Hence, it is essential to enhance the learning capacity of the network at the interface’s boundaries between two adjacent vertebrae. Giving the model as much supervision information on neighboring vertebra borders as it is physically possible to learn without incurring extra labeling costs is the optimal strategy for approaching this problem. As a result, we introduce the idea of using AVIE as one of the pathways after the backbone decoder. As shown in Figure 2d, the corner points of each vertebra are located in the sequence shown, which is clockwise. The offset is measured from the corner points ($x_{3}^{i-1}$, $y_{3}^{i-1}$), ($x_{4}^{i-1}$, $y_{4}^{i-1}$) of the upper vertebra to the corner points ($x_{1}^{i}$, $y_{1}^{i}$), ($x_{2}^{i}$, $y_{2}^{i}$) of the lower vertebra accordingly, as shown in Figure 2d. Throughout the whole training phase, the parameters of both estimators are optimized, making use of the L1 loss.

### 3.4. Vertebral Line Interpolation

**Figure 3:**
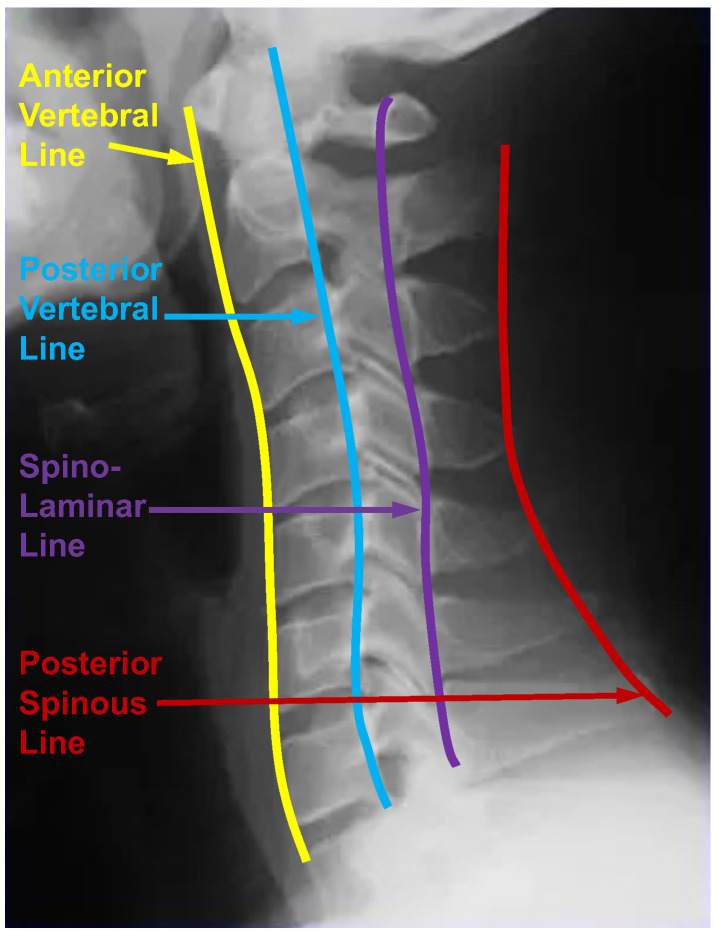
Four vertebral lines on the cervical spine image, including the anterior, posterior, spino-laminar, and posterior spinous lines.

When experts interpret cervical spine images, the alignment is crucial. They constantly assess four vertebral lines, as shown in Figure 3, which are (1) the anterior vertebral line (anterior edge of the vertebral body), (2) the posterior vertebral line (posterior edge of the vertebral body), (3) the spino-laminar line, and (4) the posterior spinous line (tip of the spinous process) [54]. These lines should adhere to a smooth, slightly anteriorly convex, non-step curve. Any misalignment will be interpreted as a sign of ligamentous damage and a hidden fracture. As the thoracic and lumbar spines are similar to the cervical spine, the vertebral line also plays an essential role in diagnosing scoliosis. As a result, the vertebral line can play an important role in assessing scoliosis. Given that scoliosis involves a change in the positions of vertebrae rather than a traumatic alteration, the vertebral line should exhibit a smooth, step-off-free curve. A line is defined as a set of infinitely extended points in opposite directions. The most straightforward way to consider the vertebral line information is to use interpolation to generate the vertebral line from the existing corner landmarks and make them the ground truth from which the model can learn. The design of ground truth is crucial for the learning in neural networks. Therefore, we optimize the ground truth design for this landmark detection task based on an intuitive understanding of scoliosis and use the optimized ground truth in the neural network training process.

**Figure 4:**
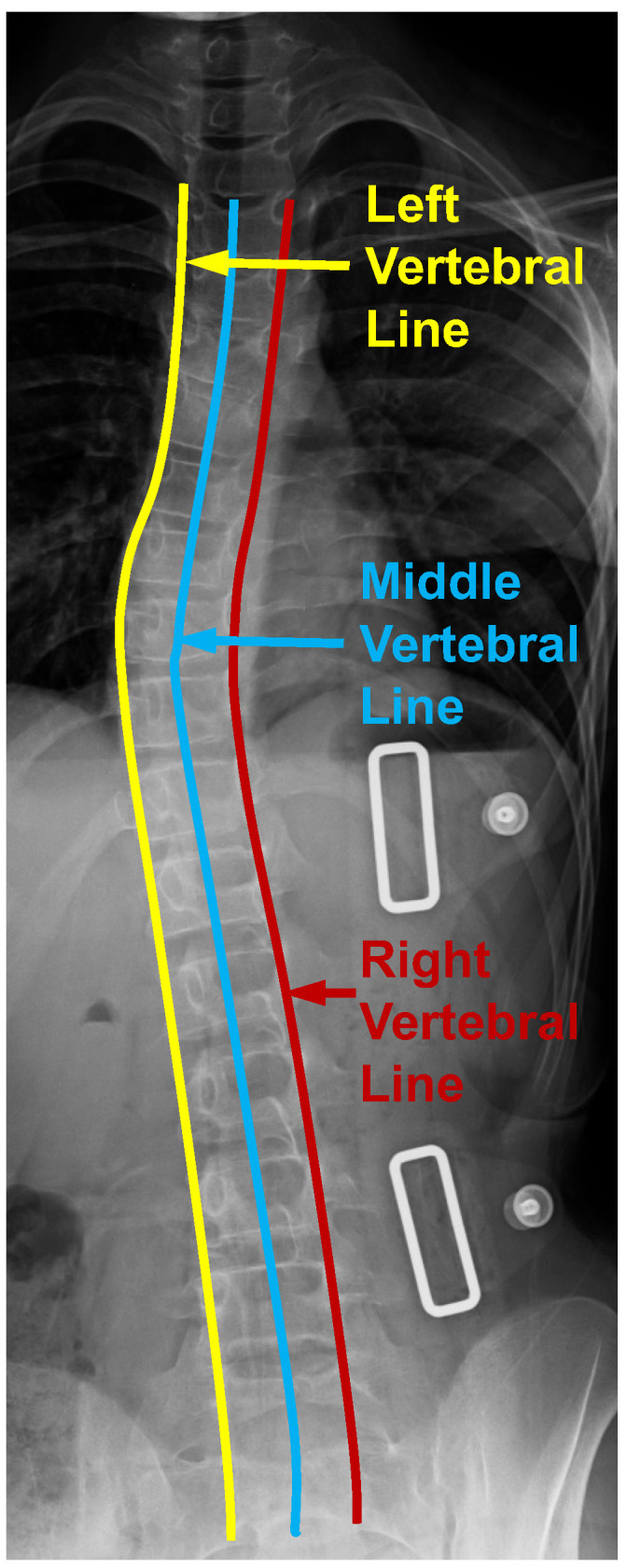
Three vertebral lines on the spinal anterior–posterior X-ray images, including the left, middle, and right vertebral lines.

From Figure 1, the vertebral line interpolation pathway in the rectangular box on the right shows how this proposed scheme works in the whole framework. Similar to the center point interval estimator pathway and the adjacent vertebra interval estimator pathway, the vertebral line interpolation pathway works as an auxiliary task in the training process. It provides additional information for better model supervision. As shown in Figure 4, we extract three vertebral lines, which include the left vertebral line, the right vertebral line, and the middle vertebral line. For the left vertebral line, we first extract all the ($x_{1}^{i}$, $y_{1}^{i}$) and ($x_{3}^{i}$, $y_{3}^{i}$) corner points. After that, we construct a b-spline curve so that all these 34 corner points are lying on this curve and then take 100 points equidistantly along this curve as the optimized ground truth. As for the right vertebral line, we first extract all the ($x_{2}^{i}$, $y_{2}^{i}$) and ($x_{4}^{i}$, $y_{4}^{i}$) corner points. The following process is the same as obtaining the left vertebral line. For the center vertebral line interpolation, first, by taking the average value of the four annotated landmarks of each vertebra, the coordinates of all center points can be obtained, which can be seen from Figure 2a. Since these 100 points from the above-mentioned three vertebral lines are very dense, we can use them as a line that can describe the overall morphology of the patient’s spine. It should be noted that the center points are obtained from four corner points representing the whole vertebra. The experimental results show that there is almost no performance difference using only the middle vertebra line. Therefore, we only used the middle vertebral line to decrease the number of parameters during the experiment. By providing extra information on the lines to the model, it is possible to enhance the accuracy of recognizing landmarks through the joint learning of points and lines. The L1 loss is used to optimize the parameters in this vertebral line interpolation pathway. The overall loss of this pathway contains three components: the left vertebral line loss, the middle vertebral line loss, and the right vertebral line loss. This is shown in Equation (3), where the subscripts *l*, *m*, and *r* indicate left, middle, and right, respectively.

(3) $$L_{VIL}=L_{vil\_l}+L_{vil\_m}+L_{vil\_r}.$$

## 4. Experiment

### 4.1. Dataset and Implementation Details

The AASCE MICCAI 2019 challenge dataset has been used to investigate our proposed method. This dataset is a publicly available dataset, which makes it possible to compare and objectively evaluate different approaches. The AASCE dataset consists of 609 anterior–posterior spinal X-ray images, which is adequate for method evaluation. An official data split is provided for fair comparison. Each X-ray image contains 17 vertebrae from the thoracic and lumbar regions of the spine. Each vertebra is annotated by four landmarks. The Cobb angle measurement procedure is determined using the algorithm presented in [55] for the AASCE dataset, which makes the evaluation more objective than only using the landmark detection error. In the experiments, we rescaled the X-ray scans to a resolution of 1024 by 512 for network input. This study used an X-ray image dataset following the data split specified in [50], comprising 60% training samples, 20% validation samples, and 20% testing samples.

All the comparison experiments were conducted on an NVIDIA 2080 graphics processing unit (GPU) and utilizing PyTorch version 1.10 for the hardware configuration. Because of the limited GPU memory, we set the batch size to 2 in all comparison experiments. For a fair comparison, we followed the training strategy in [4,50], and all models were optimized using the Adam optimizer [56]. Other weights in the network were initialized using a Gaussian distribution. Each model was trained for 150 epochs. Empirically, we set the initial learning rate to 1.25 ×$10^{-4}$. We pre-trained the ResNet34 backbone using the ImageNet dataset [57].

### 4.2. Evaluation Metrics

To statistically analyze the performance of the proposed mechanisms, the MDE values are used as the evaluation metric, which calculates the errors between the identified and ground truth landmarks. The abovementioned MDE value can be estimated as follows:

(4) $$MDE_{error}=\frac{1}{N_{total}}\sum_{j=1}^{N}{∥{\mathrm{pred}}_{j}-{\mathrm{gt}}_{j}∥}_{2},$$

where $N_{total}$ is the total number of landmarks within the dataset for testing and ${\mathrm{pred}}_{j}$ and ${\mathrm{gt}}_{j}$ represent the predicted and ground truth landmarks, respectively. The frames per second (FPS) on the NVIDIA 2080 GPU were recorded. As such, the model efficiency can be compared.

The overall MDE arises mainly from two different causes. The first one is the vertebra mismatch due to misordering. The misordering often occurs since the provided annotations have only 17 vertebrae. However, the X-ray images contain more than 17 vertebrae, including portions of the cervical and lumbar vertebrae. These redundant vertebrae can create interference and a high MDE value in certain circumstances. The other cause is less accurate localization of the corner points under accurate vertebra ordering.

To better analyze the MDE’s causes and determine the model’s strengths and weaknesses, we propose a self-adaptive MDE evaluation metric. Following the procedure in [50], we perform the top-k algorithm on the heatmap after non-maximum suppression (NMS) to obtain the coordinates of the 17 center points. Instead of fixing the value of *k* when obtaining the center points, we determine the *k* value by setting a threshold for the heatmap intensity. In our experiments, the threshold is empirically set to 0.05. This modification in setting variable *k* means that an X-ray image can be predicted with more than 17 center points. Points with intensities above a certain threshold can be interpreted as the network perceiving these points as having a high probability of being center points. Since misordering causes some cases to have a high MDE value, it leads to bias in results. Only using the MDE value makes it impossible to effectively evaluate the localization accuracy on the matched cases. We adopt the Hungarian algorithm to artificially match these vertebrae by finding the most optimal vertebra index matching between the prediction and the ground truth. After matching, the self-adaptive MDE value can be calculated according to Equation (4), similar to the MDE calculation process.

The Cobb angle is the gold standard measurement used for the scoliosis assessment [58]. In order to quantify the Cobb angle, SMAPE has been used, as shown in Equation (5). The measurements are also taken for the proximal thoracic (PT), main thoracic (MT), and thoracolumbar (TL) Cobb angles. These measurement results are referred to as SMAPE_*PT*_, SMAPE_*MT*_, and SMAPE_*TL*_, respectively. In Equation (5), *i* denotes each of the Cobb angles PT, MT, and TL, respectively. *j* refers to the image scan that is the *j*-th one to be tested, whereas $M_{total}$ indicates the total number of X-ray scans to be examined. The estimated Cobb angle and the ground truth Cobb angle are denoted by the symbols pred and gt, respectively.

(5) $$\mathrm{SMAPE}=\frac{1}{M_{total}}\sum_{j=1}^{M_{total}}\frac{\sum_{i=1}^{3}\left(\right|{\mathrm{pred}}_{ij}-{\mathrm{gt}}_{ij}\left|\right)}{\sum_{i=1}^{3}\left({\mathrm{pred}}_{ij}+{\mathrm{gt}}_{ij}\right)}.$$

### 4.3. Experimental Results

Experiments were conducted on the AASCE dataset to evaluate the detection performance of the spine’s upper, lower, and entire regions. The MDE values for corner point and center point localization using the proposed interval estimators are listed in Table 1. The results demonstrate the superior performance of these estimators in precisely identifying the center points, particularly in the lower half of the spine compared to the upper half. The MDE decreases by 10.08 pixels in the upper half and by 14.23 pixels in the lower half compared to the baseline method. The observed trend in corner point localization accuracy aligns with the motivation behind proposing these two interval estimators, as mentioned in Section 3.3. The improved performance in center and corner point localization, especially in the lower region of the spine, results in a lower SMAPE value. Specifically, the SMAPE value reduces from 9.82 to 8.61 pixels, as illustrated in Table 2.

The experimental results, as shown in Table 2, present a comparison between the baseline approach, our methods, and the final results of the ablation study. Table 2 presents the evaluation results of all 68 anatomical landmarks within the entire spine using various models. This comprehensive evaluation complements the experimental results reported in Table 1, which primarily focus on assessing the network effectiveness in specific spinal areas. We compare different evaluation metric results using (1) relative offsets from the center points to the corner points in the Cartesian coordinate system (relative), (2) the absolute Cartesian coordinates in the whole image (absolute), and (3) the relative offset from the center point in the polar coordinate system (polar). The experimental results of the baseline method are presented in the first row of Table 2, wherein only the relative Cartesian coordinates are employed for the corner points, the same as the research work by Yi et al. [50]. For the ablation study, the experimental results of six evaluation metrics were demonstrated both with and without the utilization of CPIE, AVIE, and vertebral line interpolation strategies. The data augmentation techniques used in [50] were applied to both the baseline methods and our approaches. These techniques include random cropping, expansion, contrast adjustment, and brightness distortion.

We have conducted a comparative analysis of the landmark detection performance using various interpolation methods, as listed in Table 3. Considering the well-established effectiveness of spline interpolation in reconstruction, as demonstrated in [59], we explored spline interpolation techniques beyond the conventional linear and nearest neighbor methods. Specifically, we employed zeroth-order, first-order, and b-spline interpolation methods. Our quantitative experimental findings reveal that the b-spline interpolation method outperforms others in accurately detecting vertebral landmarks.

It has been observed that the incorporation of the dual coordinate system into the proposed S2D-VLI VLDet can lead to a reduction in the SMAPE value of 1.46. At the same time, the vertebral line interpolation can boost the SMAPE value from 9.82 to 8.59. The minimum value of the MDE in Table 2 is 52.06 pixels, indicating a relatively large extent. This is mostly due to the incorrect vertebra ordering. Figure 2b shows a typical illustration. The X-ray scan reveals the presence of more than 17 visible vertebrae. However, it should be noted that only 17 vertebrae have been annotated. The lack of comprehensive annotations leads to challenges in the ordering of vertebrae and the estimation of MDE values between them since they fail to align with one another. Consequently, some cases with significant MDE can be generated. Although our proposed mechanisms can improve the accuracy of locating the center points and minimize the number of cases with a high MDE, it is impossible to eliminate such cases. Therefore, the self-adaptive MDE has been proposed to better analyze the source of the errors. As shown in Table 4, the adaptive MDE values are reduced to around 1/3–1/2 of the original MDE values (listed in Table 1) under the correct vertebra ordering. Our method improves the accuracy of corner point localization with 0.05–0.1 pixel. Although this improvement does not appear to be significant, it can be crucial to the precision of the following SMAPE value computation.

Table 5 demonstrates a comprehensive evaluation of our proposed methods in comparison to state-of-the-art approaches on the publicly available AASCE MICCAI 2019 challenge dataset. AEC-Net [60] employed a two-network architecture for landmark detection and Cobb angle estimation independently. In contrast, our approach accomplishes both tasks simultaneously using a single network. While AEC-Net achieved a SMAPE value of 23.59% for all angles, our model achieved a significantly lower SMAPE value of 8.28% on the same dataset. Another study by Yi et al. [50] utilized an encoder–decoder architecture to locate the spine’s landmarks and compute the Cobb angle, resulting in a SMAPE value of 10.81%. Seg4Reg [61] first segments the vertebrae and then directly predicts the angles, achieving a SMAPE value of 21.71%. Seg4Reg+ [62], an enhanced version of Seg4Reg [61], incorporates ResNet18 [49] as the backbone and incorporates dilated convolutions into the pyramid pooling module. The authors of Seg4Reg+ report a SMAPE value of 8.47% using ResNet18 on the same dataset.

Figure 2 shows some qualitative findings on corner offset regression and landmark detection.

## 5. Discussion and Conclusions

In this paper, we have contributed to the scoliosis assessment task through the proposed S2D-VLI VLDet network. Specifically, the sparse-to-dense vertebral line interpolation scheme, the dual coordinate system, and two auxiliary interval estimators for vertebra landmark detection have been proposed in this paper. By interpolating vertebral lines, the sparse ground truth is densified, effectively incorporating line information into the model training process. It can also retain the advantages of the Cartesian coordinate system to assist the localization process of the center points, as well as give full play to the advantages of the polar system in finding the corner point locations. The two proposed interval estimators can yield auxiliary supervision information to enhance the precision of the process for identifying the center point and corner points. In addition to the optimization in the training process, we also introduced self-adaptive MDE to evaluate the results of landmark detection more objectively, which has solved the misordering problem of vertebrae. When the aforementioned schemes are integrated into a unified framework, through the extensive experiments conducted on the AASCE dataset, we found that this novel model can improve upon the baseline landmark detection network with a convincing performance boost for both landmark detection and scoliosis assessment.

It should be noted that using b-spline interpolation to fit the vertebral line is not optimal since the vertebra is an irregular shape and its side edges are not a straight line. In the future, we can utilize self-supervised network models to localize the keypoints and fit more accurate vertebral lines as a pseudo-label without incurring additional annotation costs. In addition, the self-supervised model can work as a pre-trained model, in which the weights can be reused for the following downstream vertebra landmark detection task.

## Figures and Tables

**Table 1:** The MDE values for the vertebra center point and landmark localization of the AASCE dataset. CT, CT_*upper*_, and CT_*lower*_ represent the centers of the whole spine, the upper region of the spine, and the lower region of the spine, respectively. LM, LM_*upper*_, and LM_*lower*_ represent landmarks of the entire spine, the upper region of the spine, and the lower region of the spine, respectively. The MDE values use pixels for measuring. The downward arrows indicate that smaller values represent better model performance. Bolded experimental results denote the optimal results.

Method	Auxiliary Estimator		Corner Point Obtain			CT ↓	CT_*upper*_ ↓	CT_*lower*_ ↓	LM ↓	LM_*upper*_ ↓	LM_*lower*_ ↓
	CPIE	AVIE	Relative	Absolute	Polar						
Baseline [50]			✓			60.33	49.81	75.36	63.19	52.09	79.07
Ours	✓		✓			55.18	44.89	69.89	58.68	47.61	74.50
Ours		✓	✓			55.45	44.95	70.45	59.13	47.72	75.43
Ours	✓	✓	✓			**48.54**	**39.73**	**61.13**	**52.06**	**42.53**	**65.67**

**Table 2:** On the AASCE dataset, the table below shows the scoliosis assessment performance and vertebra landmark localization of some comparable approaches, as well as the results of the ablation study. All these MDE values are shown in mean ± standard deviation format and quantified in pixels. SP is the abbreviation for SMAPE. The downward or upward arrows indicate that smaller or larger values, respectively, signify better model performance. Bolded experimental results denote the optimal results.

Method	Auxiliary Estimator		Corner Point Obtain			Vertebral Line Interpolation	SP↓	SP_*PT*_ ↓	SP_*MT*_ ↓	SP_*TL*_ ↓	MDE ↓	FPS ↑
	CPIE	AVIE	Relative	Absolute	Polar							
Baseline [50]			✓				9.82	5.68	15.77	22.15	63.19 ±67.36	20.54
Ours	✓		✓				8.82	5.27	15.22	20.27	58.68 ±67.56	**27.72**
Ours		✓	✓				9.10	5.60	15.66	21.88	59.13 ±65.29	26.67
Ours	✓	✓	✓				8.61	**4.98**	14.59	21.48	**52.06 ±64.27**	26.56
Ours				✓			9.61	6.30	14.67	21.67	62.61 ±58.10	27.52
Ours					✓		8.36	5.04	16.21	20.21	55.61 ±63.49	12.66
Ours	✓	✓		✓			11.53	8.13	18.77	21.82	66.19 ±68.47	16.04
Ours	✓	✓			✓		8.43	5.13	**13.45**	**19.99**	53.13 ±61.15	24.67
Ours			✓			✓	8.59	5.14	13.56	20.58	59.59 ±68.10	25.11
Ours	✓	✓			✓	✓	**8.28**	5.14	14.55	20.98	56.08 ±64.34	24.68

**Table 3:** The comparison experimental results of utilizing different interpolation methods in the vertebral line interpolation pathway. Bolded experimental results denote the optimal results.

Interpolation Method	SMAPE
Linear interpolation	9.35
Spline interpolation of zeroth order	10.58
Spline interpolation of first order	10.52
Nearest interpolation	9.77
B-spline interpolation	**8.28**

**Table 4:** Experimental results of the self-adaptive MDE values. The abbreviations CT, CT_*upper*_, CT_*lower*_, LM, LM_*upper*_, and LM_*lower*_ are the same as in Table 1. Bolded experimental results denote the optimal results. The downward arrows indicate that smaller values represent better model performance.

Method	CT ↓	CT_*upper*_ ↓	CT_*lower*_ ↓	LM ↓	LM_*upper*_ ↓	LM_*lower*_ ↓
Baseline [50]	17.83	12.38	25.62	22.32	15.96	31.40
CPIE+AVIE+relative	18.32	**12.28**	26.95	22.93	**15.91**	32.97
CPIE+AVIE+polar	17.94	12.39	25.88	23.16	16.26	33.02
CPIE+AVIE+polar+vertebral line interpolation	**17.79**	12.37	**25.54**	**22.27**	15.95	**31.30**

**Table 5:** Comparison with state-of-the-art methods on the public AASCE MICCAI 2019 challenge dataset.

Methods	SMAPE	PT	MT	TL	MSE
Khanal et al. [63]	26.05	-	-	-	-
Wang et al. [41]	23.43	26.38	30.27	35.61	77.94
Chen et al. [60]	23.59	-	-	-	-
Yi et al. [50]	10.81	6.26	18.04	23.42	50.11
Horng et al. [35]	16.48	9.71	25.97	33.01	74.07
Dubost et al. [64]	22.96	-	-	-	-
Wang et al. [65]	12.97	-	-	-	-
Lin et al. (ResNet18) [62]	8.47	-	-	-	-
Guo et al. [66]	8.62	4.76	15.83	21.04	52.72
Ours	8.28	5.14	14.55	20.98	56.08

## Data Availability

The AASCE dataset is a publicly available dataset. The official homepage is https://aasce19.grand-challenge.org/ 3 January 2024.
